# The Role of *Cutibacterium acnes* in Sarcoidosis: From Antigen to Treatable Trait?

**DOI:** 10.3390/microorganisms10081649

**Published:** 2022-08-15

**Authors:** Raisa Kraaijvanger, Marcel Veltkamp

**Affiliations:** 1 Interstitial Lung Diseases Centre of Excellence, Department of Pulmonology, St. Antonius Hospital, 3435 CM Nieuwegein, The Netherlands; 2 Division of Hearth and Lungs, University Medical Center Utrecht, 3584 CX Utrecht, The Netherlands

**Keywords:** *Cutibacterium acnes*, immune modulation, sarcoidosis

## Abstract

*Cutibacterium acnes* (*C. acnes*, formerly *Propionibacterium acnes*) is considered to be a non-pathogenic resident of the human skin, as well as mucosal surfaces. However, it also has been demonstrated that *C. acnes* plays a pathogenic role in diseases such as acne vulgaris or implant infections after orthopedic surgery. Besides a role in infectious disease, this bacterium also seems to harbor immunomodulatory effects demonstrated by studies using *C. acnes* to enhance anti-tumor activity in various cancers or vaccination response. Sarcoidosis is a systemic inflammatory disorder of unknown causes. Cultures of *C. acnes* in biopsy samples of sarcoidosis patients, its presence in BAL fluid, tissue samples as well as antibodies against this bacterium found in serum of patients with sarcoidosis suggest an etiological role in this disease. In this review we address the antigenic as well as immunomodulatory potential of *C. acnes* with a focus on sarcoidosis. Furthermore, a potential role for antibiotic treatment in patients with sarcoidosis will be explored.

## 1. Introduction

*Cutibacterium acnes* (formerly *Propionibacterium acnes*) (*C. acnes*) is an anaerobic-aerotolerant Gram-positive, rod shaped bacterium and commensal of the human skin, oral cavity, large intestine, conjunctiva and the external ear canal [1,2,3]. The fact that the human defense system allows *C. acnes* to colonize the body on several levels suggests that this bacterium, at least under normal circumstances, is not harmful for the human host. Based on serology, two phenotypes of *C. acnes* have been distinguished: type I and type II. Later on, phylogenetic analysis based on nucleotide sequencing of a nonribosomal housekeeping gene (recA) and a putative hemolysin/cytotoxin gene (tly) showed that type I and type II represented different groups and that type I could be subdivided in type IA1, IA2, IB and IC [4,5]. More recently, a third phenotype was identified and was designated as type III [6]. These different *C. acnes* strains differ in their induction of proinflammatory cytokines and their ability to induce an immune responses [2].

## 2. *C. acnes* and Different Diseases

**Figure 1:**
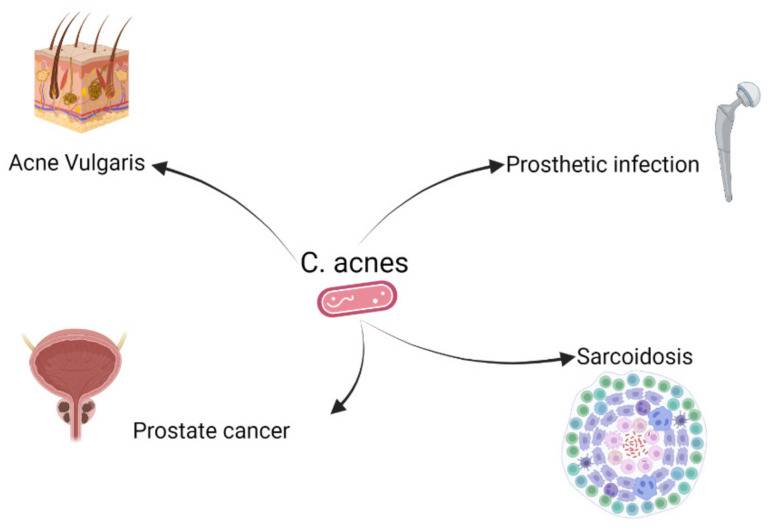
Known and suspected disorders associated with Cutibacterium acnes. There is convincing scientific evidence that *C. acnes* is involved in the development of acne vulgaris and prosthetic infections. Furthermore, associations have been found between the occurrence of prostate cancer or sarcoidosis and the presence of *C. acnes*, for which a causal role has not yet been demonstrated.

Traditionally*, C. acnes* has been considered as a non-pathogenic resident of the human microbiota. Over the years, however, *C. acnes* has also been suspected to plays a role in various disorders such as acne vulgaris (Figure 1). Already in 1896 *C. acnes* was associated with acne vulgaris and now its etiological role is widely accepted, even though the precise mechanisms are yet to be fully elucidated [2]. *C. acnes* has a prominent part in the normal human skin flora, where it plays a critical role in the regulation of skin homeostasis and preventing colonization of other harmful pathogens [2,7]. Comparing healthy skin with skin affected by acne vulgaris revealed that there was no difference in the load of *C. acnes* [8]. Based on these data it was hypothesized that inflammatory skin disorders may not result from *C. acnes* acting as an opportunistic pathogen, but rather the imbalance in skin microbiota may play a critical role [7,8]. Treatment of acne vulgaris comes with complications. Current treatments are often difficult to tolerate or are inadequate to inhibit the inflammatory effect. Isotretinoin, for example, is a treatment extensively prescribed for the treatment of severe acne, but can cause depression and comes with an increased risk of birth defects when taken during pregnancy [9,10]. Moreover, the use of antibiotics as treatment for acne vulgaris may result in resistance development of the *C. acnes* bacterium [11]. In 2018, Wang et al. proposed the use of a vaccination with the *C. acnes* Christie-Atkins-Munch-Petersen (CAMP) factor, a virulence factor triggering cell death, to potentially decrease *C. acnes*-induced inflammation [12]. With the use of a mouse model it was shown that this vaccination increased protective immunity against *C. acnes*. In mice, a decreased *C. acnes* colonization and reduced production of a proinflammatory MIP-2 cytokine was shown after vaccination [12].

*C. acnes* not only seems to play a role in inflammation of the skin but also in infections of the heart, the gastrointestinal tract and also in orthopedic surgery, particularly in relation to implants [3,13,14]. In the last decade, an increased number of studies have found an association between *C. acnes* and a wide range of implant-associated infections (IAI) [15]. *C. acnes* is the cause of various post-surgical associated infections, including breast implants [16,17], ocular implants, neurosurgical shunts [18], spinal hardware, cardiovascular devices [13] and prosthetic joints [19]. Of all implant-associated infections caused by *C. acnes*, shoulder prosthetic joints are most often infected. This is probably due to greater colonization in the shoulder and armpit than for example in the hip or knee [14,20]. Once *C. acnes* is settled on the prosthesis it can form a biofilm, which needs to be fully eliminated in order to cure the infection. The formation of a biofilm is a pertinacious trait and often requires the full replacement of all implants [20].

Moreover, it has also been shown that *C. acnes* is associated with the inflammation of the prostate as well as prostate cancer. A study from Sweden showed that *C. acnes* could be cultured in 60% of the prostate cancer cases and in 26% of the cancer-free controls [21], whereas a French study detected only very few *C. acnes* positive samples in their cohort [22]. However, based on the biopsy procedure involving antibiotic prophylaxis, these results are difficult to interpret. It is now hypothesized that intraepithelial *C. acnes* infection in non-cancerous prostate glands resulting in excessive inflammation may contribute to the development of prostate cancer. Furthermore, in patients diagnosed with prostate cancer, this infiltration is associated with a worse prognosis [23,24,25].

## 3. Immunomodulatory Effect of *C. acnes*

### 3.1. Anti-Tumor Effect

In addition to the fact that *C. acnes* can be seen as an infectious agent, it is also capable of modulating the immune system resulting in an anti-tumor activity against various animal and human cancers [26,27,28]. The anti-tumor effect of *C. acnes* has been reported in multiple studies of various types of cancer, such as malignant melanoma [29] and breast cancer [28]. However, *C. acnes* is not the only bacterial strain which has an anti-tumor and immune-modulatory effect. Several bacteria such as *Clostridium novyi* [30], *Bifidobacterium longum* [31] and *Salmonella typhi* [32] have also been shown to have this effect.

**Figure 2:**
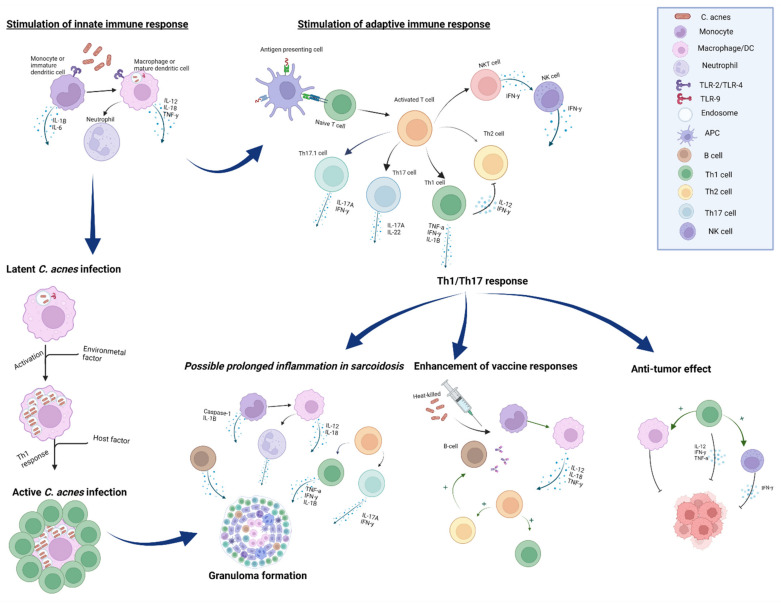
Proposed interaction between Cutibacterium acnes and the immune system resulting in different immunoregulatory effects. *C. acnes* has extensive immunostimulatory activity. It can stimulate different innate immune receptor such as TLR-2, TLR-4 and intracellular TLR-9 and NOD-like receptors. *C. acnes* can induce a Thelper-1 (Th1) cytokine milieu and subsequent Th1 response which is thought to be beneficial in enhancing both anti-tumor response and vaccination. Furthermore, *C*. *acnes* seems capable of skewing a Th2 response to a Th1 response. Recent data also suggest that *C*. *acnes* is involved in initiating a more Th17-based response. The intercellular *C. acnes* infection is thought to induce granuloma formation in sarcoidosis. The mechanism of this antigenic role of *C. acnes* was hypothesized by Eishi et al. (adapted with permission from [66]). However, *C. acnes* is also thought to play a mitogenic role to granuloma formation in sarcoidosis, mainly based on induction of a Th1/Th17 response.

*C. acnes* can modulate a variety of cellular functions of lymphocytes and macrophages which have been shown to be beneficial for an anti-tumor response (Figure 2). An important example of such modulation is the induction of a Th1 response, resulting in the activation of macrophages and natural killer (NK) cells [33,34,35]. Moreover, it has also been shown that *C. acnes* is able to skew a Th2 response towards a Th1 response. A Th2 environment is favorable in tumor formation based on the fact that there is less activity of cytotoxic T-cells and NK cells [36]. Using a mouse model for melanoma and breast cancer it was shown that *C. acnes* infection resulted in a switch in the immune response toward a Th1 response, both locally in the tumor as well as in the peripheral blood compartment. This resulted in an increased infiltration of T-cells into the tumor lesions of these mice [28,29]. Upon activation by the *C. acnes*, NK cells infiltrate the tumor after being attracted by tumor-secreted cytokines and cell-death signals. Dendritic cells, in their turn, leave the cancer tissue and migrate to the draining lymph nodes, where they present captured antigens to naïve and memory T-cells [37]. The phagocytosis of *C. acnes* by dendritic cells results in a Th1-type cytokine immune response and causes the production of IFN-γ, TNF-α and IL-12 [28].

The anti-tumor effect is not induced by all types of *C. acnes*. Mouse model studies showed that the injection of *C. acnes* type I resulted in survival of 60 to 100% of the cases, while type II did not show this same anti-tumor effect [38,39]. The difference in anti-tumor activity might be explained by the difference in the carbohydrate composition of the cell walls of the different types of *C. acnes* resulting in different immunological and biological activity [40]. Moreover, the anti-tumor effect of type I strains might also be due to the more potent resistance of the strains to degradation by phagocytes than type II strains [41]. Interestingly, *C. acnes* not only showed an anti-tumor effect, but also showed an anti-metastatic effect by inhibiting the spread and growth of metastasized tumor cells [42].

### 3.2. C. acnes as a Vaccine Adjuvant

Based on its immunomodulatory capacity*, C. acnes* has also been studied regarding a potential beneficial role as a vaccine adjuvant in infectious disease (Figure 2). Due to the cytokine pattern mediated by *C. acnes*, its heat-killed suspension has been used as a Th1 response inducer to enhance the efficacy of a HIV vaccine [43]. It was hypothesized that the *C. acnes* modulation of the immune system probably occurs by direct action on antigen-presenting cells (APCs), such as macrophages, DCs and B lymphocytes [44].

In a mouse model, the soluble polysaccharide fraction of *C. acnes* also induced an enhanced vaccination response in experimental *T. cruzi* infection [45].

In equine respiratory infections, inactivated *C. acnes* is commercialized under the name of Neogen^®^Vet EqStim^®^ (Neogen Coroporation, Lansing, MI, USA) and administered as either prophylactic treatment or to enhance recovery from respiratory infections [46,47]. A study showed a significant improvement in the recovery as well as a significant decrease in disease severity from respiratory diseases in horses treated with heat-killed *C. acnes* compared to untreated controls [48]. These results were later confirmed by a second study treating 25 horses with *C. acnes* of which 96% of the horses showed complete recovery or improvement compared to 35% of the control group [47]. When entering the blood stream, *C. acnes* is phagocytized by macrophages in the liver or spleen. The degradation of *C. acnes* is delayed and could be the reason for the immune-modulatory properties of this bacterium [46,49,50].

In all vaccination studies it appears that the induction of a Th1 response by *C. acnes* in particular is the most important factor for the adjuvant properties of this bacterium.

### 3.3. Innate Immunity

As mentioned previously, *C. acnes* can interact with cells of the immune system on different levels (Figure 2). Not only macrophages, but also lymphocytes and neutrophils react to the pathogenic factors released by *C. acnes*, such as proteases, lipases and chemotactic factors [51]. Interaction with *C. acnes* and the immune system results in the stimulation and production of anti-microbial peptides and various chemokines and cytokines [52,53,54]. Toll-like receptors (TLR) on the immune cells can recognize *C. acnes* both extracellularly and intracellularly. It was seen that these immune cells were directly activated via TLR-2 and TLR-9 [55,56]. *C. acnes* is able to survive intracellularly inside macrophages, enabling it to evade the immune response of the host [57]*. C. acnes* produces catalase in order to facilitate this intracellular localization. Furthermore, *C. acnes* has a cell wall with a high resistance to degrading and oxidizing enzymes, enabling intracellular persistence [58].

### 3.4. Adaptive Immunity

Initially, *C. acnes* was seen as an Th1-inducing agent due to the cytokine pattern that is induced, such as IL-1α, IL-6, TNF-α, IL-12 and IL-18, which result in the release of IFN-γ [44,59]. Furthermore, early inflammatory acne lesions were shown to have an activated Th1 lymphocyte response [60].

In addition, *C. acnes* was shown not only to induce the production of Th1-type cytokines, but also Th17-type cytokines, such as IL-17. IL-17 is a pro-inflammatory cytokine which plays a role in various autoimmune and inflammatory disorders [61]. However, Th17 cells were shown to play a role in protecting the host against extracellular bacteria and fungi [62].

In recent years it has become clear that *C. acnes* can promote a mixed Th17/Th1 response by inducing the secretion of IL-17A and IFN-γ. When studying the blood of patients suffering from acne vulgaris it was seen that both Th17 and Th17.1 cells were found in higher frequencies than in healthy individuals and may play a possible role in its pathogenesis [63,64]. In this disease, the Th17 cells were shown to be related to the activation of CD4+ Th-cells resulting in an increase in secreted IL-17 in acne-related strains [64,65]. The differentiation of Th subsets was shown to be tightly regulated by transcription factors, signaling transducers and activator of transcription (STAT). STAT4 was shown to regulate Th1 differentiation, whereas Th2 differentiation is dependent on STAT5a. STAT3 in its turn is responsible for the differentiation of Th17 differentiation [62]. As mentioned previously, *C. acnes* was also shown to have an adjuvant effect by modulating the immune system. This adjuvant effect is related to a direct effect of the bacterial components on APCs, especially on dendritic cells. When the bacterial components bind the APCs expression of costimulatory molecules and TLRs is modulated, which was shown to be essential for driving the T-cell response [44,59]. Moreover, B1 cells were also shown to play a role in the adjuvant effect of *C. acnes*. B1 cells are a sub-class of B cell lymphocytes and their importance in adaptive immune responses has been well established.

## 4. The Role of *C. acnes* in Sarcoidosis

### 4.1. Sarcoidosis and Possible Antigens

Sarcoidosis is a multi-systemic granulomatous disorder which most often affects the lungs and peripheral lymph nodes, but the disease can also affect other organs [67,68]. In sarcoidosis so called ‘epithelioid cell granulomas’ are formed by a Th1/Th17.1 immune response against an antigen with a strong antigenicity [69]. The lung is constantly confronted with airborne substances including micro-organisms. As this organ is most often affected by sarcoidosis, many researchers have considered infection as a trigger and have tried to identify possible infectious agents in sarcoidosis [70]. Over the years, many possible infectious but also noninfectious agents such as metals or silica have been suggested, summarized in Table 1. Regarding infectious agents, most studies were performed addressing the possible role of either *mycobacteria*, *aspergillus* species or *C. acnes* as antigens in sarcoidosis [71].

### 4.2. C. acnes as Antigen in Sarcoidosis

In the past decades, multiple studies have suggested that *C. acnes* might be a disease-causing antigen in a subgroup of patients with sarcoidosis. First of all, *C. acnes* has sporadically been cultured from the lymph nodes of patients with sarcoidosis [77,99]. However, as *C. acnes* is a commensal, Koch’s postulates cannot be applied, making it difficult to interpret these findings [100].

Secondly, *C. acnes* has been detected by immunohistochemical (IHC) staining in the tissues of various organs of patients with sarcoidosis, including the lungs [77,101,102,103], lymph nodes [77,101,104], skin [101] and heart [105].

In the case of the identification of the antigen in granulomas, possible antigens are more likely to be found in immature granulomas with many inflammatory cells than in mature granulomas in which the degradation process is almost completed and only a few lymphocytes are still present.

Thirdly, the DNA of *C. acnes* was shown to be amplified in a higher percentage of sarcoidosis patients than in the DNA of mycobacterium tuberculosis [106]. This was confirmed by Zhou et al., who combined data from various studies using meta-analysis and found that 78% of patients with sarcoidosis had *C. acnes* within the lesions [107]. However, the detection of DNA can also point to latent infection unrelated to the formation of sarcoidosis. This could be remedied with the use of a quantitative PCR, which can discriminate between a potential latent infection and a reactivated infection [71].

Finally, *C. acnes* elicits a specific immunoglobulin response in sarcoidosis patients. Increased local immunoglobulin production, especially IgG and IgA concentrations, were detected in BAL of sarcoidosis patients [108,109]. Schupp et al. were the first to show a B-cell-specific immune response to *C. acnes* in Caucasian sarcoidosis patients and showed that IgA production was increased only in early disease, especially Löfgren syndrome, while IgG was elevated in chronic disease as well [108]. Previously, Japanese studies already reported an increased Th1 immune response and increased IgG and IgA titers in PBMCs from sarcoidosis patients with presence of *C. acnes* [110,111,112].

Beijer et al. [88] tried to establish trigger-related phenotypes in Dutch sarcoidosis patients by measuring immunological sensitization with the use of enzyme-linked immune absorbent spot (ELISpot) assays. In case of immunological sensitization for *C. acnes*, 3.5% of the sarcoidosis patients showed a positive ELISpot for *C. acnes* catalase. Looking at the clinical phenotype, patients with a positive ELISpot for *C. acnes* catalase showed a trend towards more cutaneous involvement of the sarcoidosis and were significantly younger at diagnosis compared to patients without a positive *C. acnes* catalase ELISpot [84]. However, contrary to what was previously found in the immunoglobulin response studies [108,110,111], the immunological sensitization of *C. acnes* was much lower in Dutch sarcoidosis patients compared to the control group of obstructive sleep apnea (OSA) patients (3.5% versus 15%). Beijer et al., suggested that this lower percentage could reflect the homing of the cells to the lungs. However, it is not clear why the T cell response was lower for the sarcoidosis patients [88]. Based on these results one may argue that in order to study *C. acnes* sensitization in sarcoidosis, peripheral blood may not be the correct compartment.

### 4.3. A Possible Role of C. acnes as a Mitogen in Sarcoidosis

Traditionally, in the quest for the cause of sarcoidosis, the main focus has been the antigenic potential of microorganisms or inorganic materials studied. However, studies on the role of *C. acnes* have been hampered by the fact that this bacterium is a human commensal and indeed was found to be the most common commensal in lung tissue and mediastinal lymph nodes in patient without sarcoidosis [113]. In previous studies detecting *C. acnes* in sarcoidosis, available lung tissue after resection for lung cancer or granulomatous tissue of patients with active mycobacterial infection were used as a controls [67].

**Figure 3:**
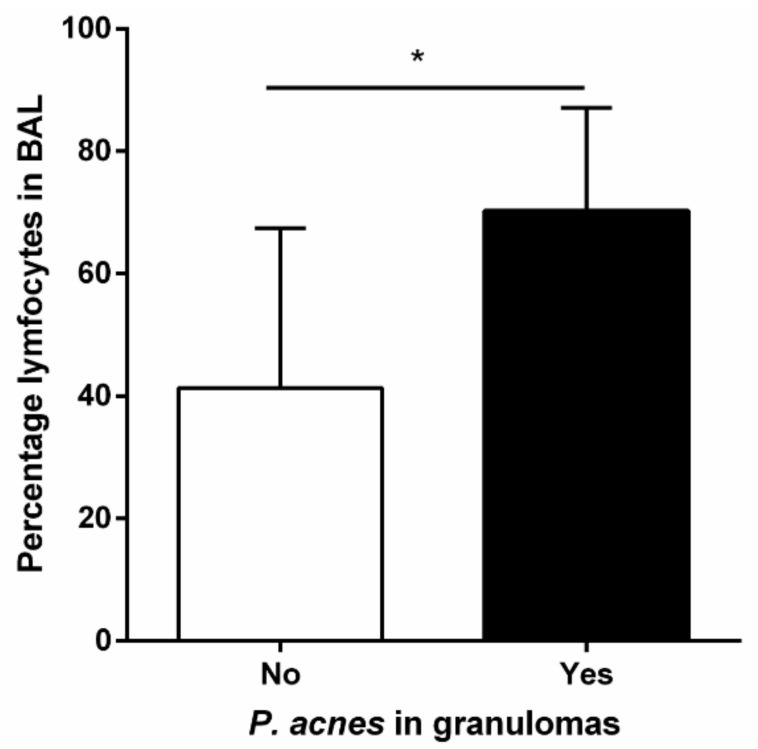
Lymphocyte percentage in the BAL fluid of HP patients with and without the presence of *C. acnes* in granulomas. Asterisk represents significant difference. BAL: Broncho alveolar lavage, HP: Hypersensitivity pneumonitis. The results of this study are described in [114].

Data on whether *C. acnes* could also be present in non-infectious granulomatous pulmonary diseases such as hypersensitivity pneumonitis (HP) or vasculitis were not available. However, recently Beijer et al. demonstrated that C. acnes could also be detected in the granulomatous tissue of patients with HP and Eosinophilic granulomatosis with polyangiitis (EGPA). Interestingly, in light of the potential of *C. acnes* to skew an immune response towards a more Th-1 response they categorized HP patients into a group where *C. acnes* was present in the granulomas and a group without the presence of *C. acnes* in the granulomas. When comparing the amount of lymphocytosis in BAL fluid between both groups it was found that the presence of *C. acnes* in the granulomas of patients with HP was associated with a significantly higher amount of lymphocytes in BAL fluid (Figure 3), further supporting the hypothesis that the role of *C. acnes* could be mitogenic in different granulomatous pulmonary disorders [114].

## 5. Can the Presence of *C. acnes* in Patients with Sarcoidosis Be Seen as Treatable Trait?

Unfortunately, there is still no curative treatment for sarcoidosis [68]. Immunosuppressive drugs, such as steroids or disease-modifying antirheumatic drugs (DMARDs) are often prescribed aiming for a reduction in symptoms and the prevention of organ damage. However, when using immunosuppressive therapy, patients often experience many burdensome side effects [115,116,117]. There is a need for more personalized treatment in sarcoidosis, which could potentially be accomplished by identifying (non)infectious agents in individual sarcoidosis patients. In the case of *C. acnes*, one could speculate that a subgroup of sarcoidosis patients might benefit from therapy with antibiotics. The potential clinical relevance was already suggested in a study using a mouse model: in a group of 14 mice, 4 demonstrated parenchymal non-caseating granulomas after the mice were sensitized for *C. acnes*. However, if these mice were pre-treated with azithromycin, known to be bactericidal to *C. acnes*, only one of the mice developed a small number of granulomas after sensitization with this bacterium [118].

Moreover, for sarcoidosis patients there are already multiple case reports describing successful treatment with antibiotics of *C. acnes*-related sarcoidosis, especially in Japanese sarcoidosis patients. In 2008, a case of a 47-year-old female suffering from sarcoidosis affecting several parts of the body was described in whom treatment with minocycline resulted in an reduction in muscular sarcoidosis and a significant decrease in the serum Angiotensin-convertering enzyme (ACE) level [119]. Furthermore, in 2014 a case of a 78-year-old woman with *C. acnes*-associated sarcoidosis was described in which treatment with clarithromycin was shown to induce a resolution of the sarcoid granulomas and was found to be effective for treatment of the sarcoidosis in this particular patient [120]. However, no randomized controlled trials investigating the potential effect of antibiotic treatment in a patient with sarcoidosis where *C. acnes* is actually present in their granulomatous tissue have been carried out. In 2018 the J-Acnes trial was initiated, where patients with cardiac sarcoidosis are treated with antibacterial drugs in addition to the immunosuppressive therapy [121]. The results of this trial are eagerly awaited.

**Figure 4:**
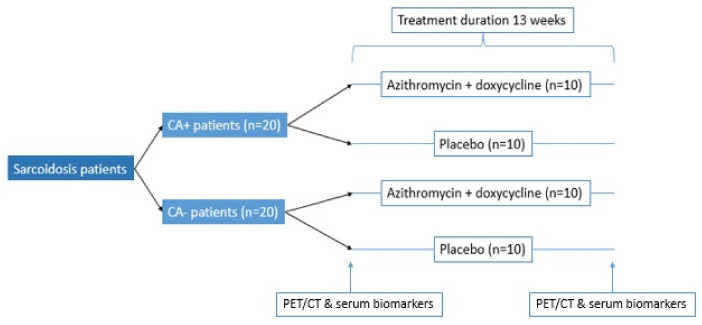
Flow chart of the PHENOSAR trial. Patients are stratified based on the presence or absence of Cutibacterium acnes in their granulomas. After this stratification, patients are randomized to receive either 500 mg Azithromycin 3 times a week and daily doxycycline, or a placebo for 13 weeks. CA+ = sarcoidosis patients with the presence of *C. acnes* in granulomatous tissue. CA− = sarcoidosis patients with no *C. acnes* in their granulomatous tissue. PET/CT = Positive Electron Tomography/Computer Tomography.

Recently, the PHENOSAR study was initiated in 2022 (ClinicalTrials.gov, Identifier: NCT05291468). This will be the first trial in which targeted therapy in sarcoidosis is based on the presence of *C. acnes* in granulomatous tissue. In this trial a combination of two antibiotics, azithromycin and doxycycline, will be used to minimize the chance of resistance development of the *C. acnes* bacterium. A total of four groups will be formed which will receive either antibiotics or a placebo, as shown in Figure 4.

Patients will receive treatment/placebo for 13 weeks after which the burden of inflammation is determined by comparing PET/CT-scan and serum biomarkers ACE and IL-2R of baseline and 13 weeks. Hopefully this study will provide data in order to determine whether the presence of *C. acnes* in patients with sarcoidosis could be seen as a treatable trait for this disease.

## 6. Conclusions

The human commensal *C. acnes* seems to be a jack of all trades with regard to possible influences on innate immunity, anti-tumor response, enhancement of vaccine effectivity and etiology of various diseases such as acne vulgaris, prostate cancer and sarcoidosis.

Over the past decades *C. acnes* has been identified as a possible cause of sarcoidosis; however, the discussion about causality vs. association with this disease is still not settled. Recent data suggest that *C. acnes* might also have a more mitogenic role in sarcoidosis but future studies are definitely needed to clarify the exact role of *C. acnes* in the pathogenesis of sarcoidosis.

## Figures and Tables

**Table 1:** Possible antigens in sarcoidosis.

Possible Antigens	**Literature (References Are in Parentheses)**
Infectious agents	
*Mycobacterium tuberculosis*	[72,73,74,75]
*Cutibacterium acnes*	[76,77]
*Borrelia*	[78]
*Fungi*	[79,80,81]
*Aspergillus nidulans*	[82,83]
Non-infectious environmental antigens	
Beryllium	[84,85,86,87,88]
Aluminum	[84,88,89]
Silica	[85,88,89,90]
copper	[91]
titanium	[85,89,91,92]
Combustible wood	[93,94,95]
Autoantigens	
vimentin	[96,97,98]

## Data Availability

The study did not report any data.
